# Frequency of appearance of transverse (Harris) lines reflects living conditions of the Pleistocene bear—*Ursus ingressus*—(Sudety Mts., Poland)

**DOI:** 10.1371/journal.pone.0196342

**Published:** 2018-04-23

**Authors:** Dariusz Nowakowski

**Affiliations:** Department of Anthropology, Wrocław University of Environmental and Life Sciences, Wrocław, Poland; University of Warsaw, POLAND

## Abstract

Transverse lines, called Harris Lines (HL), osteological markers of recovery from growth arrest episodes, are visible in radiograms of recent and Pleistocene fossil bones. Since on the one hand they mark stressful episodes in life, and on the other are mainly used to trace health fluctuations in prehistoric human communities, I used a cave bear population to check if the processes that could affect the specie’ condition were in any way reflected in the bone structure. 392 bear bones from Bear Cave in Kletno (collection: Department of Palaeozoology, University of Wrocław), dated as 32 100 ±1300 to >49 000 years BP, were radiologically examined. The bones were found in a non-anatomical position; morphological analysis indicated that they belonged to different individuals. HL shadows were observed on 9 tibiae and 3 radii: 8.8% out of the 59 tibiae and 77 radii and 3.1% of all the bones. At least 3 transverse lines were recognised in those cases; the specimens were histologically examined. The bear individuals in question experienced regular malnutrition periods during their ontogeny. Starvation resulting in growth inhibition involved young individuals, aged 1 to 4 years. Juveniles aged 6 months, i.e. before weaning, or younger, showed no signs of nutritional stress. Starvation periods associated with seasonal food deficit were not long or common and had no significant effect on the development and welfare of the species. This is the first description of the occurrence of transverse lines in the Pleistocene bear.

## Introduction

Wegner [1] studied fossil human bones and observed lines of increased tissue density.

The interest in those lines grew with the advent of the Roentgen technique, and first attempts were made to explain such a pathological picture in human bones. Initially, it was thought to be associated with diet-induced disturbances in bone growth which led to scar formation in the spongy tissue [2].

The lines may be of different shape, thickness and course. Different definitions were proposed in the course of studies on transverse lines. Initially they were defined as single lines, not less than 5 mm long, extending from the diploe toward the middle of the bone shaft [3]. According to Clark [4] the line should be visible on radiograms and extend at least to half of the bone shaft, while in the opinion of Goodman and Clark [5] the line should be characterised by a distinct contrast as a result of increased density of the bone substance on at least 1/4 of the shaft. However, the observations of transverse lines show that their picture varies even within the same bone [6], so that each of the above definitions can be regarded as correct.

Harris [7] explained their origin by inhibited bone growth; since then they were called Harris lines. The Harris lines were mainly analysed when describing health condition and diet of human population, as one of indicators of stress in its broad sense [2,8]. It was found that their formation was an individual reaction of organism to physiological disturbances [9]. It was thus accepted that transverse lines in long bones provided much information on the ontogenetic and population phenomena [10,11]. The investigations of the Harris lines expanded the knowledge of the organism’s reaction to adverse environmental conditions. Considerations on the origin of transverse lines in recent, historic and fossil, populations contributed to the knowledge of the range of factors which cause delays in bone growth. According to Steinbock [12] such factors include acute inflammations, periods of starvation, food composition deficits, poisoning and other pathogenic factors. The mechanism of origin of transverse lines was more precisely described by researchers who experimented on animals [13,14]. The Harris lines appeared in bones of animals with protein or vitamin deficit [15,16]. Their occurrence was most often described in human bones. In experimental studies HL were found in some mammals and birds: dog [7], rat [17], rabbit [1,15], chicken [1], and pig [18]. Papers dealing with fossil animals are few. Duckler and Valkenburgh [19] described HL in late Pleistocene mammals: dire wolf *(Canis dirus)*, coyote (*C*. *latrans)*, sabertooth cat *(Smilodon fatalis)*, bison *(Bison antiquus)*, camel *(Camelops hesternus)*, and horse *(Equus laurentius)*. The authors suggest that the late Pleistocene species were not suffering from unusual levels of poor health.

The extensive cave bear material from Kletno (SW Poland) provided a unique opportunity to examine the occurrence and possibly the origin of HL in a population of the species.

## Material and methods

Following the preliminary analysis of ca. 40 000 bones and bone fragments of *Ursus ingressus* from Bear Cave in Kletno, 392 (humerus, radius, ulna, femur, tibia, fibula) were selected for further studies. They represented different individuals whose number was impossible to estimate.

The specimens, found in the deposits of Bear Cave in Kletno (Sudety Mts., Poland) are kept in the collection of the Palaeozoology Department, Wrocław University.

The bone remains from Bear Cave were radiocarbon dated as 32 100±1 300 to >49 000 years BP [20]. All the dates correspond to the period (MIS 4—MIS 2) from the Świecie Stadial—Grudziądz Interstadial to the main stadial of the Vistula Glaciation (LGM) [20,21].

The bones were subject to analysis with mutually supplementing X-ray and histological methods. X-ray photos were taken with digital technique (focus-object distance 1m) in a-p norm. The occurrence of HL was analysed based on the X-rays. Then the images of the lines and the outlines of the analysed pictures were transferred to tracing paper as 1:1 and measured to the nearest 0.1 mm. The measurements served as the basis for further analyses.

**Fig 1:**
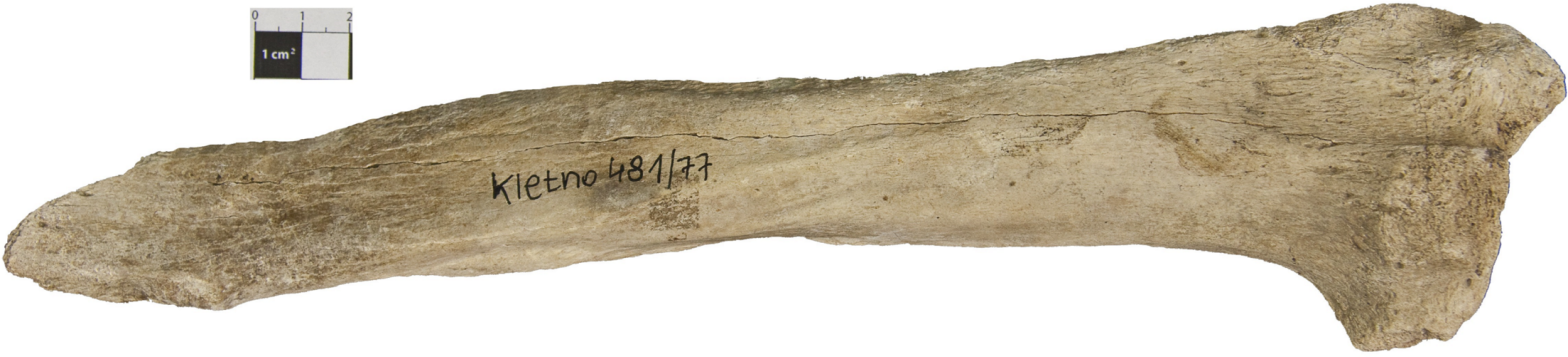
Bone fragment (radius) N° 481/77.

**Fig 2:**
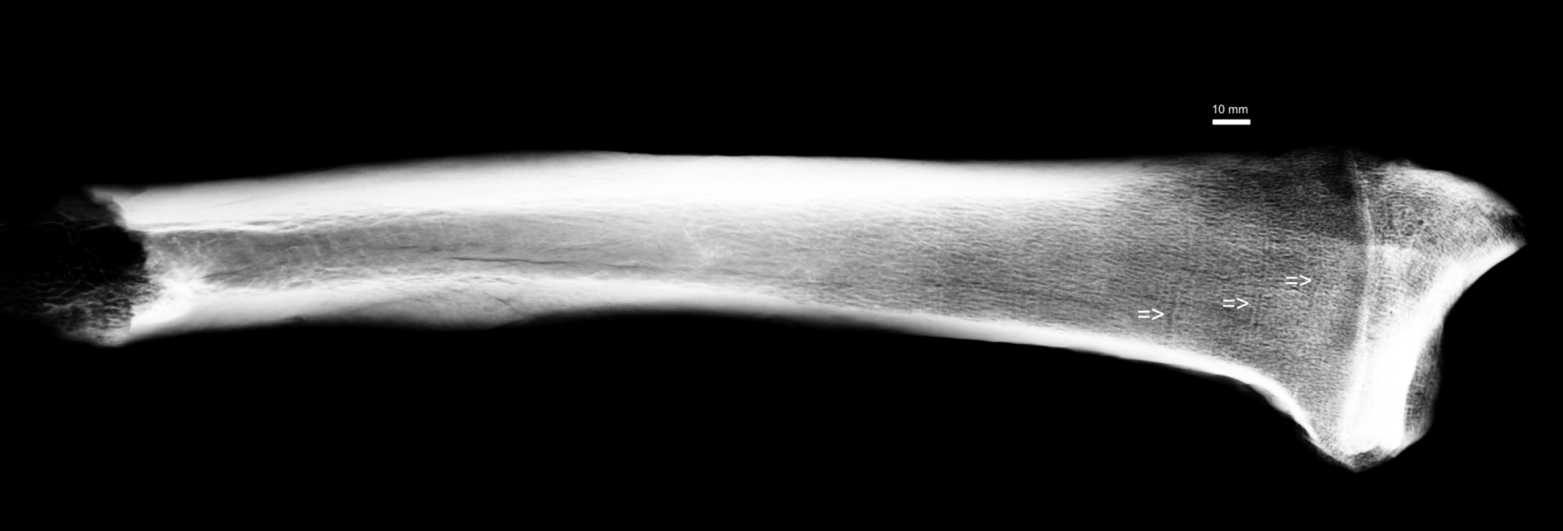
X-ray of radius N° 481/77. Harris Lines indicated with arrows.

**Fig 3:**
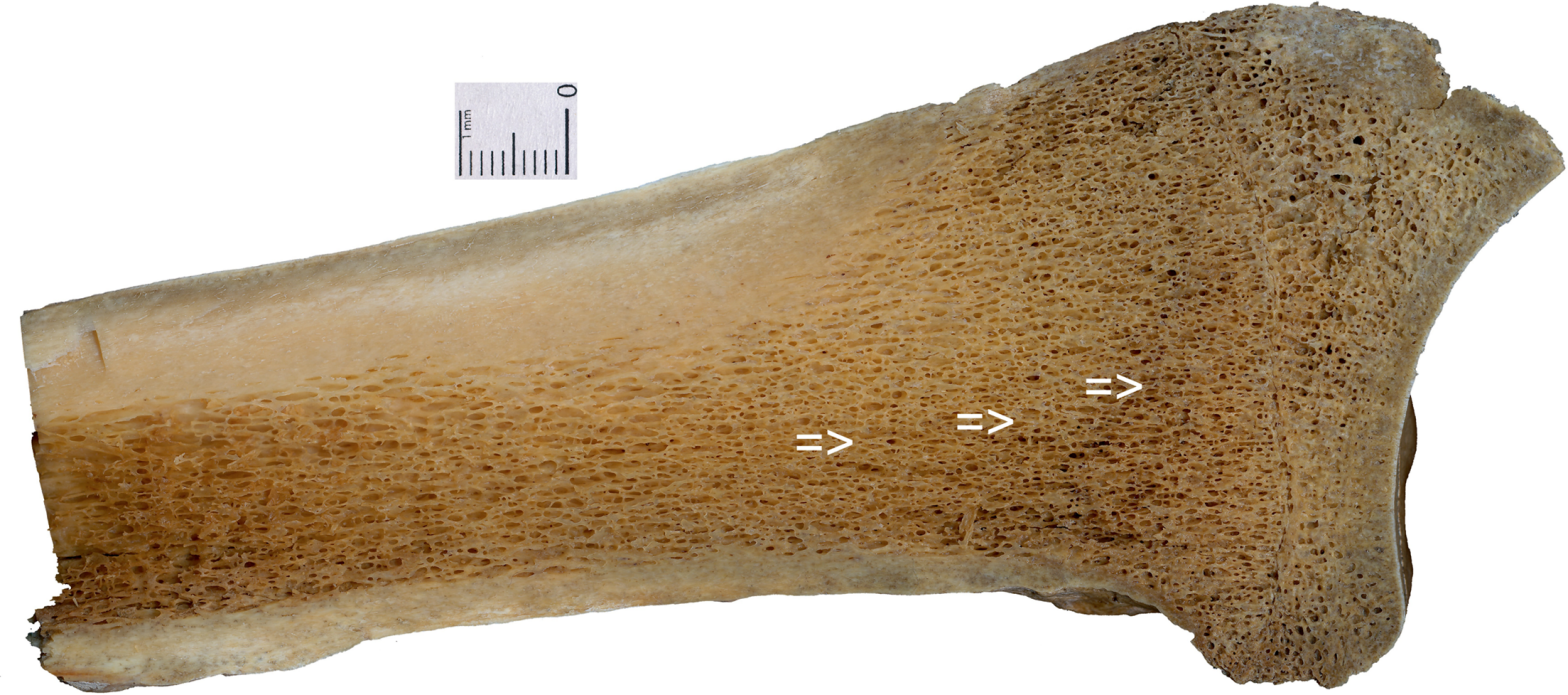
The cross-section of radius N° 481/77. Area with HL, Harris Lines indicated with arrows.

Bone fragment N^o^ 481/77 (Fig 1), X-ray (Fig 2), in order to visualise the spongy tissue structure with transverse lines (Fig 3, arrows) was cut out with a diamond saw Bauer GMBH and histologically examined as a bone section in light microscope [embedded in Epon Sigma-Aldrich Chemie GmbH].

## Results

The shadow of transverse lines identified as Harris lines was observed on 9 tibiae and 3 radii: 8.8% out of the 59 tibiae and 77 radii and 3.1% of all the bones.

Among the observed cases of the Harris lines, on the radiograms of all the bones at least three transverse lines were recognised. Most frequently, i.e. in 9 cases, 4 lines were found. In one case 6 lines were observed, and in one bone transverse lines were observed on both epiphyses (Table 1). No transverse lines were observed on the radiograms of bones of very young individuals i.e. bones with no epiphyses preserved, because of incomplete ossification. All the observed Harris lines were delicate and, apart from their length, they showed no significant structural differences. The observed length of the lines varied depending on the kind of bone, even within the same specimen, from 8 to 41 mm. All the lines were situated at a distance not longer than 54 mm from the epiphysis margin. On the radiograms the intervals between the lines were regular.

**Table 1:** Distribution of transverse lines observed on long bones.

N^o^	bone	extrem.	k0/d	k1/d	k2/d	k3/d	k4/d	k5/d	k6/d
**692**	*tibia*	*distalis*	13/50	19/30	24/34	31/17	39/ 9	-	-
**K-528**	*tibia*	*distalis*	9/ 56	18/24	26/24	33/14	43/ 6	-	-
**K-513**	*tibia*	*distalis*	16/56	22/15	26/16	34/29	43/20	-	-
**K-I/886**	*tibia*	*distalis*	10/55	20/32	25/25	33/29	40/19	-	-
**671**	*tibia*	*distalis*	8/ 49	14/ 9	26/28	34/22	41/13	47/15	-
**693**	*tibia*	*distalis*	9/ 50	23/31	28/32	37/11	-	-	-
**672**	*tibia*	*distalis*	11/23	17/ 9	22/ 7	29/11	33/10	40/ 8	56/12
**709**	*tibia*	*distalis*	13/42	21/24	30/32	37/19	-	-	-
**K-8/465**	*tibia*	*proximalis*	20/65	24/27	29/30	38/41	49/27	61/23	-
**K-8/465**	*tibia*	*distalis*	10/30	15/8	19/22	27/20	34/11	-	-
**K-342/77**	*radius*	*proximalis*	13/55	20/13	28/21	40/23	54/19	-	-
**695**	*radius*	*proximalis*	15/48	22/34	33/18	53/ 9	-	-	-
**481/77**	*radius*	*proximalis*	15/59	27/16	37/29	55/25	-	-	-

**Fig 4:**
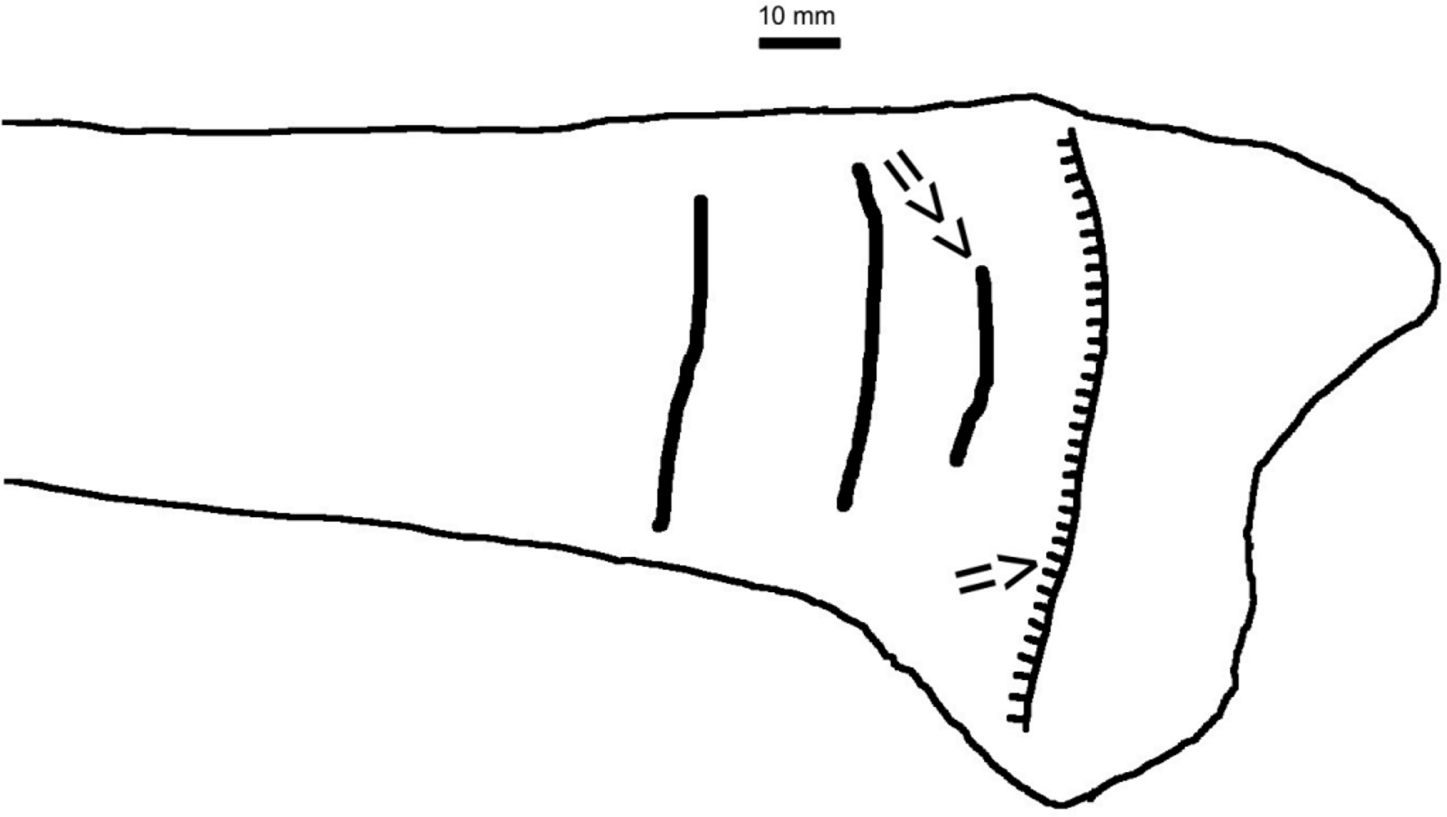
Scheme of radius N° 481/77. Shadow of line ossification nucleus—arrow = >; shadow of first Harris line—arrow = >>).

Distances between: k0—shadow of line ossification nucleus and epiphysis margin; k1—shadow of first line ossification nucleus and epiphysis margin, k2—shadow of second line ossification nucleus and epiphysis margin, k3—shadow of third line ossification nucleus and epiphysis margin, k4—shadow of fourth line ossification nucleus and epiphysis margin, k5—shadow of fifth line ossification nucleus and epiphysis margin, k6—shadow of sixth line ossification nucleus and epiphysis margin; /d–length of line measured as a straight line, distance between extreme points (e.g. bone N^o^ 481/77, cf. Fig 4).

**Fig 5:**
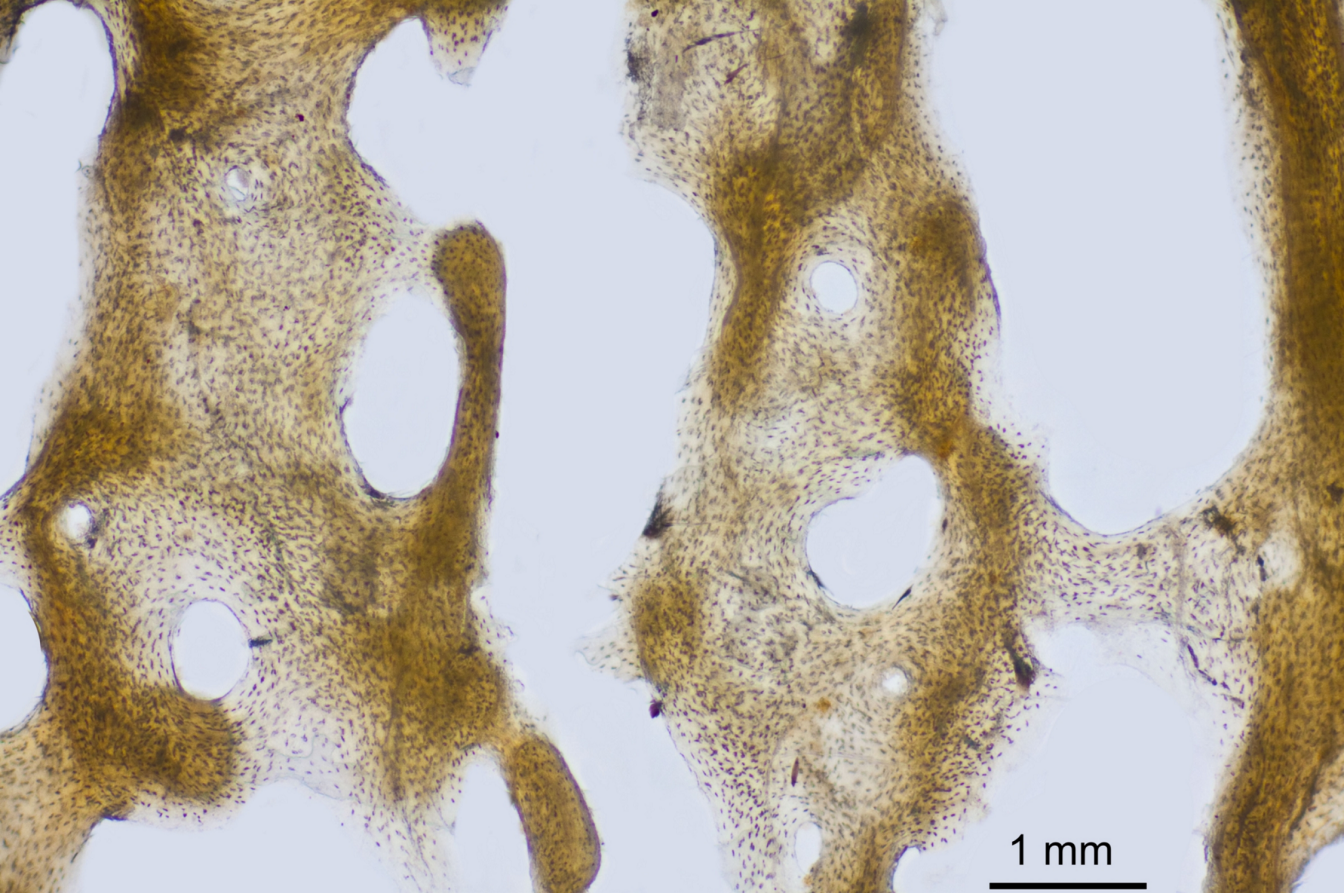
Area of spongy tissue with HL.

The cross-section of radius No 481/77, in the area of HL the spongy tissue is less compact (Fig 3, arrows). The size of the osteons and Haversian canals caniculi, and the number of bone cells in these areas, are similar (Fig 5).

## Discussion

The detailed analysis of the radiogram pictures of bone structure disturbances in the form of transverse lines (Table 1) classified them as pathology called the Harris lines.

In the examined material transverse lines appeared only in two kinds of long bones: tibia and radius (Table 1). The lines observed in the proximal and distal para-diaphysis parts of tibia and the distal para-diaphysis part of radius are compatible with the results of other authors [12] who found that such lines were common in those places, though their occurrence in other parts of long bones was also possible.

The different length of the observed sclerotic areas (Table 1) is probably associated with resorption of the lines during the individual’s lifetime. A complete resorption of some, probably present earlier, transverse lines explains the small disturbances in the distances between the lines. Probably, considering the possibility of resorption of the lines in adult life, the regularity of their distribution should be more marked and observable in all cases.

The distances between the lines and their number, seen in particular specimens (Table 1), suggest that the stress factor acted regularly during the bone growth [4]; the factor acted periodically twice a year.

The thickness of transverse lines is affected by the duration of bone growth inhibition, its rate and the fact that the Harris lines may run obliquely to the shaft and be incomplete [22]. The situation may be age-dependent [3]. Such re-structuring of bone tissue could have arisen only before completion of bone longitudinal growth, and the resulting lines get resorbed a few years after their formation. Gran and Baby [3] found that the lines disappeared with age, at a rate dependent on the bone mineralisation processes. Some of the lines remain visible till senility [12]. In mammals, a period of starvation as short as two weeks can initiate the process of bone restructuring [14,23]. Given that the incidence of Harris lines is associated with relatively severe stress in at least one mammal population, the presence of the lines in late Pleistocene mammals could be used to support hypotheses of elevated stress levels. Research of Duckler and Valkenburgh [19] shows that probably it provides no support for such hypotheses. On the examined bones, all the transverse lines are delicate and show no structural differences, suggesting a small intensity of the growth inhibiting factor [24].

The small incidence of Harris lines in *Ursus ingressus* from Bear Cave may also result from the fact that the examined population came from long before the extinction of *Ursus ingressus*; it is not possible to conclude about the dependence between the HL and the species’ extinction. Most of the dates point to MIS 3. Even the locality Jaskinia Stajnia, with the youngest remains of the species [25], includes MIS 4 and MIS 3. A more extensive material from various sites should no doubt be examined. It is known, however, that the end of MIS 4 and MIS 3 were periods of frequent and dynamic climate fluctuations which affected the natural environment including vegetation, and thus the cave bear condition.

The closest extant relative of *Ursus ingressus*, which occurred also at the same time as the cave bear is the brown bear *Ursus arctos* L. Its distribution range includes, among other areas, the northern regions of Europe and Asia with their climate resembling that of the distribution range of the Pleistocene bear [26]. It is thus possible to compare the behaviour and growth rate of the species, as well as environmental factors which affected them [26–29]. The comparison suggests that food deficits occurred twice a year: in spring and summer. It can be supposed that the starvation periods were associated with seasonal food deficits and preceded periods of intense feeding. Only during adaptation to independent life were young individuals exposed to starvation periods which resulted in growth inhibition and formation of transverse lines on their bones.

Transverse lines form a pattern which reflects pathologies of the skeleton of individual at different stages of its life [22]. The distances between the lines and their distance from the epiphysis indicate at which period of life the bone growth was inhibited [2]. The pattern of lines makes it possible to ascertain the moment of effect of stress factor in relation to the individual’s age. Since the longitudinal growth rates of the bones is an individual character, it would be advisable to use a few parameters, for example bone age and tooth wear, to determine the animal’s age [30]. The examined material does not permit age estimate since no articulated skeletons were found in Bear Cave. Despite this approximate age of the bones was established based on the earlier-described methods.

The absence of transverse lines on the bones of cave bear less than one year in age indicates that they underwent no stress which would cause bone growth disturbances while still in full care of the mother. It can be supposed that very young individuals were not exposed to starvation periods which would cause stress, and such seasonal food deficits in the cave bear life occurred only after weaning at the age of one year, when the mother’s milk stopped being sufficient for the cub’s growth and the bear had to forage on its own. The pattern of lines showed also that the lines were formed when the bone size was typical of pre-adult animals [27] and the growth-inhibiting factor was active till the completion of epiphysis ossification, that is the age of 3–4 years.

In his studies of transverse lines McHenry [23] detected no statistically significant sex-related differences in the frequency of the lines. Likewise, there is no statistically significant dependence between the frequency of the Harris lines and the adult bone length [22]. Thus, the individual size which is correlated with bone length and sex should have no effect on the described disturbances in the bone growth of cave bear.

## Conclusion

Though the studies on bone structure disturbances in the form of Harris lines are rendered difficult by an array of associations with individual morphological characters and with the environment, it is possible to assess the health condition of fossil populations based on transverse lines on the bones [31]. Based on the examined material it can be said that only few individuals of the cave bear population from the Sudety Mts. were exposed to cyclic starvation periods during their ontogeny. The starvation occurred in the period preceding spring, as a result of hibernation, and was associated with increased food demand in the autumn, as in the extant brown bear [32]. Starvation periods leading to bone growth inhibition probably involved only young individuals, between the first and fourth year of life. The cave bear individuals underwent no starvation stresses till the age of half year, since they were fed with mother’s milk. When the young individual started foraging for plant food on its own it became exposed to starvation periods which caused stress. It can also be supposed that the starvation periods were not long. Since transverse lines were observed only in 3.1% of the examined material, the starvation periods in the examined population were infrequent and had no significant effect on the development and growth of the species during the Pleistocene. This confirms the thesis of Duckler and Valkenburgh [19], that physiological stress resulting in formation of transverse lines is not correlated with any significant decrease in condition which could affect the population’s mortality during the Pleistocene.

The description of transverse lines presented here is probably the first record of their occurrence on the bones of *Ursus ingressus*.
